# The Effect of Depression on Smartphone Addiction: The Medicating Effects of Interpersonal Problem in Korean Adolescents

**DOI:** 10.1192/j.eurpsy.2024.314

**Published:** 2024-08-27

**Authors:** Y. Seo, Y. Nam, J. Lee

**Affiliations:** Department of Psychiatry, Yonsei University Wonju College of Medicine, Wonju, Korea, Republic Of

## Abstract

**Introduction:**

Problematic smartphone use is twice as common among teenagers as it is among adults. Smartphone addiction is associated with anxiety, depression, attention deficit disorder, impulsivity, and sleep problems, among other issues.

**Objectives:**

To assess whether interpersonal relationship problems mediate the relationship between smartphone addiction and depression among adolescents (ages 12-17) currently enrolled in middle and high school.

**Methods:**

A cross-sectional study was conducted among 653 middle and high school students living in Wonju, South Korea between September 1 and November 30, 2019. Depression was measured by Center for Epidemiologic Studies Depression Scale (CES-D). In order to evaluate smartphone addiction, the Smartphone Addiction Scale Short Form Version (SAS-SV) was used. To examine interpersonal problems, the Korea Inventory of Interpersonal Problems Circumplex scale (KIIP-SC) was employed. We used the dplyr package to check for skew, kurtosis, and create density plots. Scatterplots and Pearson correlation analysis were used to examine the relationships between the main variables. For the mediation analysis, we used the 8 sub-scales of KIIP-SC (Domineering, Vindictive, Cold, Socially avoidant, Nonassertive, Exploitable, Overly Nurturant, Intrusive) as mediators and conducted a mediation analysis with 10,000 bootstrap samples using the lavaan package in R, version 4.2.2. Each analysis was evaluated based on a 95% confidence interval to determine significance.

**Results:**

Depression, interpersonal problems, and smartphone addiction exhibited significant positive correlations with each other. The direct effect of smartphone addiction was found to be significant. The association between depression and smartphone addiction was mediated by the KIIP-HI (Nonassertive), the KIIP-JK (Exploitable) and the KIIP-NO (Intrusive).

**Conclusions:**

Interpersonal problems mediate the relationship between depression and smartphone addiction. Identifying the high-risk group is essential for treatment strategy development.

**Disclosure of Interest:**

None Declared

